# Potential Novel Biomarkers for Diabetic Testicular Damage in Streptozotocin-Induced Diabetic Rats: Nerve Growth Factor Beta and Vascular Endothelial Growth Factor

**DOI:** 10.1155/2014/108106

**Published:** 2014-03-20

**Authors:** Ali Rıza Sisman, Muge Kiray, Ulas Mehmet Camsari, Merve Evren, Mehmet Ates, Basak Baykara, Ilkay Aksu, Guven Guvendi, Nazan Uysal

**Affiliations:** ^1^Department of Biochemistry, Dokuz Eylul University, 35340 Izmir, Turkey; ^2^Department of Physiology, Dokuz Eylul University, 35340 Izmir, Turkey; ^3^Division of Behavioral Physiology, Department of Physiology, School of Medicine, Dokuz Eylul University, 35340 Izmir, Turkey; ^4^Department of Neuroscience, Mayo Clinic, Jacksonville, FL 32224, USA; ^5^Department of Psychiatry, Mayo Clinic Health System, Waycross, GA 31501, USA; ^6^College of Natural and Applied Sciences, Department of Biotechnology, Ege University Bornova, 35920 Izmir, Turkey; ^7^College of Vocational School of Health Services, School of Medicine, Dokuz Eylul University, 35340 Izmir, Turkey; ^8^College of Physical Therapy and Rehabilitation, Dokuz Eylul University, Balcova, 35340 Izmir, Turkey

## Abstract

*Background*. It is well known that diabetes mellitus may cause testicular damage. Vascular endothelial growth factor (VEGF) and nerve growth factor beta (NGF-**β**) are important neurotrophic factors for male reproductive system. *Objective*. We aimed to investigate the correlation between testicular damage and testicular VEGF and NGF-**β** levels in diabetic rats. *Methods*. Diabetes was induced by streptozotocin (STZ, 45 mg/kg/i.p.) in adult rats. Five weeks later testicular tissue was removed; testicular VEGF and NGF-**β** levels were measured by ELISA. Testicular damage was detected by using hematoxylin and eosin staining and periodic acid-Schiff staining, and apoptosis was identified by terminal-deoxynucleotidyl-transferase-mediated dUTP nick end labeling (TUNEL). Seminiferous tubular sperm formation was evaluated using Johnsen's score. *Results*. In diabetic rats, seminiferous tubule diameter was found to be decreased; basement membrane was found to be thickened in seminiferous tubules and degenerated germ cells. Additionally, TUNEL-positive cells were increased in number of VEGF+ cells and levels of VEGF and NGF-**β** were decreased in diabetic testes. Correlation between VEGF and NGF-**β** levels was strong. *Conclusion*. These results suggest that the decrease of VEGF and NGF-**β** levels is associated with the increase of the apoptosis and testicular damage in diabetic rats. Testis VEGF and NGF-**β** levels could be potential novel biomarkers for diabetes induced testicular damage.

## 1. Introduction

Diabetes mellitus is the most common chronic endocrine metabolic disorder [1]. Diabetes causes many functional and structural complications in different organs, such as testis, pancreas, and brain [2–4]. Diabetes can impair male reproductive functions in both humans and animals [5, 6]. Diabetes also impairs spermatogenesis and reduces sperm count, sperm motility, seminal fluid volume, and testosterone levels [3, 5, 6]. In our previous study, we showed that seminiferous tubule diameter was reduced and basement membrane was thickened in seminiferous tubules and degenerated germ cells in diabetic animals [3].

Vascular endothelial growth factor (VEGF) is known as neurotrophic and angiotrophic factor; therefore, it induces proliferation of endothelial cells and increases permeability of the vessel wall [7, 8]. Sertoli and Leydig cells both produce VEGF and have VEGF receptors [7]. VEGF is important in germ cell homeostasis [9].

NGF is a neurotrophic factor that regulates a number of vital functions of the neurons including survival, growth, proliferation, and differentiation [10]. NGF is found in the seminal vesicle, epididymis, testis, Leydig cells, Sertoli cells, and spermatogonia [11–13]. It stimulates sperm motility and facilitates sperm cell acrosome reactions [14]. In addition, NGF is important for the proliferation and differentiation of Leydig cells and NGF promotes testosterone production [15]. These previous studies indicate that both VEGF and nerve growth factor (NGF) play an important role for male reproductive system [7–9, 11–13, 15].

The aim of this study is to investigate the correlation between testicular damage and testicular VEGF and NGF-*β* levels in diabetic rats.

## 2. Materials and Methods

Adult male Wistar Albino rats (Dokuz Eylul University, Experimental Animal Laboratory, Izmir, Turkey) were housed in individual cages with free access to water and laboratory chow. Rats were maintained in a 12 h light/12 h dark cycle at constant room temperature (22 ± 1°C), humidity (60%). All experimental procedures were performed as approved by the Animal Care and Use Committee of the Dokuz Eylul University, School of Medicine.

**Figure 1:**
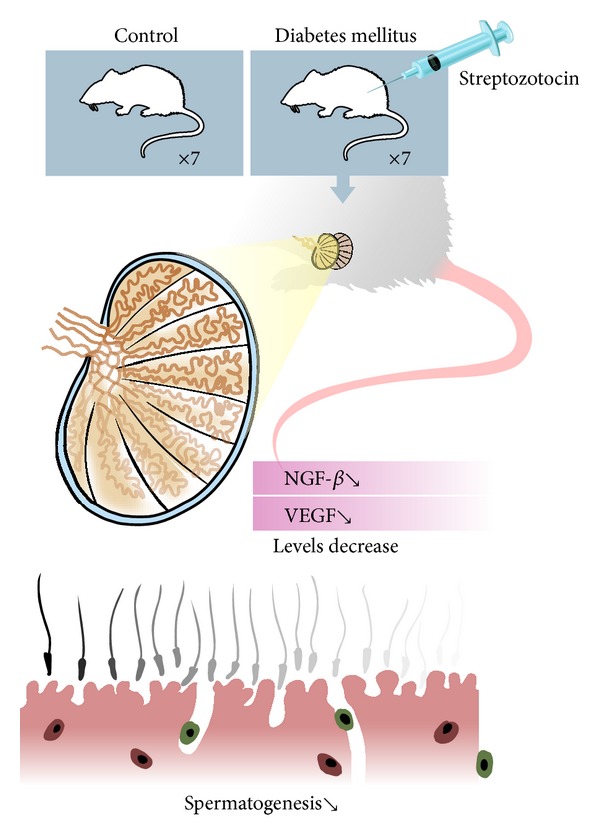
Representative picture of experiment.

Rats were divided into two groups: (1) control group (*n* = 7) and (2) diabetic group (*n* = 7). Diabetes was induced by a single intraperitoneal injection of streptozotocin (Sigma, St. Louis, MO; 45 mg/kg) (Figure 1). Twenty-four hours after streptozotocin treatment, induction of diabetes in the experimental group was confirmed by blood glucose levels over 250 mg/dL [4, 16].

Five weeks after streptozotocin injection, following a light ether anesthesia, the testes tissues were extracted for biochemical and histological examination.

Testes tissue samples were fixed in 10% formalin in phosphate buffer for 24 h. The tissues were sectioned into sequential 5 *μ*m sections using a microtome (Thermo Finesse M+). All sections were stained by hematoxylin-eosin and periodic acid-Schiff (PAS). The images were analyzed using a computer assisted image analyzer system consisting of a microscope (Olympus CX-41 Tokyo, Japan) equipped with a high-resolution video camera (Olympus DP21, Japan).

For PAS staining, the sections were incubated in 0.1% periodic acid for 5 min. The slides were washed in running tap water and immersed in Schiff's reagent for 15 min. Subsequently, the sections were washed in tap water for 10 min, counterstained with Mayer's hematoxylin, washed in tap water, and dehydrated in graded ethanol. Finally, the sections were cleared in xylene and mounted with Entellan.

### 2.1. Examination of Spermatogenesis

Johnsen's score was used to categorize the spermatogenesis [14]. It applies a grade from 1 to 10 to each tubule cross section according to the following criteria: 10 = complete spermatogenesis and perfect tubules; 9 = many spermatozoa present and disorganized spermatogenesis; 8 = only a few spermatozoa present; 7 = no spermatozoa but many spermatids present; 6 = only a few spermatids present; 5 = no spermatozoa or spermatids but many spermatocytes present; 4 = only a few spermatocytes present; 3 = only spermatogonia present; 2 = no germ cells but only Sertoli cells present; 1 = no germ cells and no Sertoli cells present.

### 2.2. Measurement of Seminiferous Tubule Diameter

The 10 most circular seminiferous tubules were randomly identified in each section of the testis, and their diameters were measured with an ocular micrometer using the 40x objective. The mean seminiferous tubule diameter (MSTD) in micrometers was determined for each testis.

### 2.3. Measurement of Seminiferous Tubule Basement Membrane

Five-micrometer-thick sections were obtained from each animal and they were stained with PAS. All sections were viewed under a microscope with an attached video camera and image analyzer system (CellSens Entry). The measurement was performed on 10 randomly selected seminiferous tubule basement membranes (STBM) from each section and averaged.

### 2.4. Measurement of Apoptosis

Apoptosis was evaluated by the in situ terminal-deoxynucleotidyl-transferase-mediated dUTP digoxigenin nick end labeling (TUNEL) assay. TUNEL staining was performed using an In Situ Cell Death Detection Kit (Roche, Germany) according to the manufacturer's protocol. Briefly, the sections were deparaffinized, hydrated by successive series of alcohol, washed in distilled water followed by phosphate-buffered saline (PBS), and deproteinized by proteinase K (20 *μ*g/mL) for 15 min at 37°C. Then the sections were rinsed and incubated in the TUNEL reaction mixture. The sections were rinsed and visualized using converter-POD with 0.02% 3,3′-diaminobenzidine (DAB). The sections were counterstained with hematoxylin. Detection of apoptotic cells was performed under the light microscope at a magnification of 40x. The apoptotic index was defined as the number of apoptotic TUNEL-positive cells per 100 tubules. Two observers blinded to the source of testicular tissue performed all measurements.

### 2.5. VEGF Immunohistochemistry

Immunohistochemical staining of testis tissues was performed using the streptavidin/biotin method (85-9043, Invitrogen, Camarillo, CA). The immunohistochemistry procedure for VEGF (SC-7629, Santa Cruz, USA) was performed. Tissue sections were incubated at 60°C overnight then dewaxed in xylene for 30 min. After rehydrating through a decreasing series of alcohol, sections were washed in distilled water for 10 min. They were then treated with 10 mM citrate buffer (AP-9003-125, Labvision) at 95°C for five minutes, to unmask antigens by heat treatment. Then slides washed in deionized water three times for two minutes. Sections were incubated in 3% hydrogen peroxide for 10 min to inhibit endogenous peroxidase activity. They were then incubated with normal serum blocking solution for 30 minutes. Sections were incubated in a humid chamber with antibody to VEGF (1/50 dilution: SC-7629, Santa-Cruz Biotechnology). For negative controls, distilled water was used in place of the primary antibody. They were washed three times for 5 min each with PBS, followed by incubation with biotinylated IgG and then with streptavidin-peroxidase conjugate. After washing three times for 5 min with PBS, sections were incubated with DAB substrate containing diaminobenzidine for 5 min to detect immunoreactivity and then with Mayer's hematoxylin. Sections were covered with mounting medium. Immunohistochemical evaluation was performed based on the intensity of VEGF immunoreactivity in the testes. A semiquantitative immunolabelling scale from 1 to 4 was graded as follows: 1, none; 2, mild; 3, moderate; and 4, strong.

### 2.6. Biochemical Investigation

VEGF and NGF-*β* levels of testes homogenates were measured using commercially available ELISA kits specific for rat (VEGF Catalog number EK0308, Boster Immunoleader, Wuhan, China with assay sensitivity <1 pg/mL and range 15.6–1000 pg/mL; NGF-*β*, Catalog number EK0471, Boster Immunoleader, Wuhan, China, with assay sensitivity <1 pg/mL and range 15.6–1000 pg/mL), according to the manufacturer's instructions.

### 2.7. Statistical Investigation

Statistical analysis was performed by using SPSS 15.0. Differences between groups were calculated using a nonparametric test (Mann Whitney *U* test). Correlations among groups were calculated using Pearson correlation analysis. Results are presented as mean ± S.E.M. *P* < 0.05 was considered statistically significant.

## 3. Results

**Figure 2:**
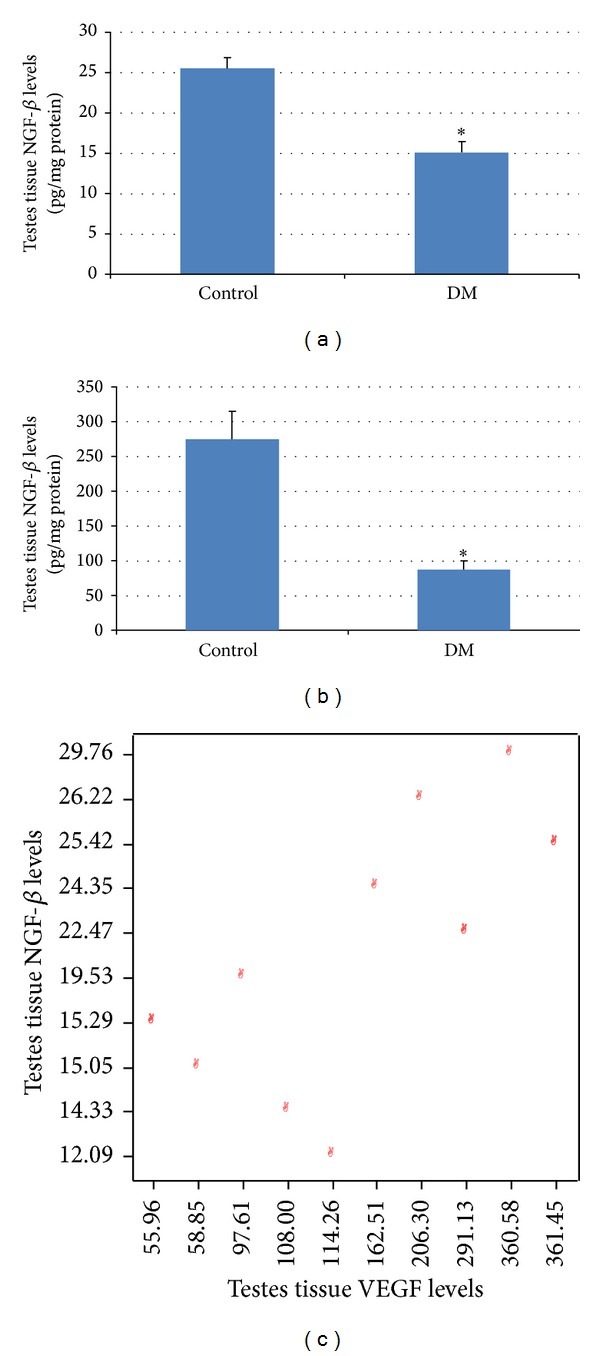
Biochemical investigation results: (a) testis NGF-*β* levels and (b) testis VEGF levels. (c) Correlation between NGF-*β* and VEGF levels of testis, (7 control, 7 diabetic rats), *n*: 14, *r* = 0.925, *P* = 0.0001, *P < 0.05, DM: diabetes mellitus.

Diabetic process was found to be correlated with decreased VEGF and NGF-*β* levels in testicular tissue (both of *P* < 0.009) (Figures 2(a) and 2(b)).

**Figure 3:**
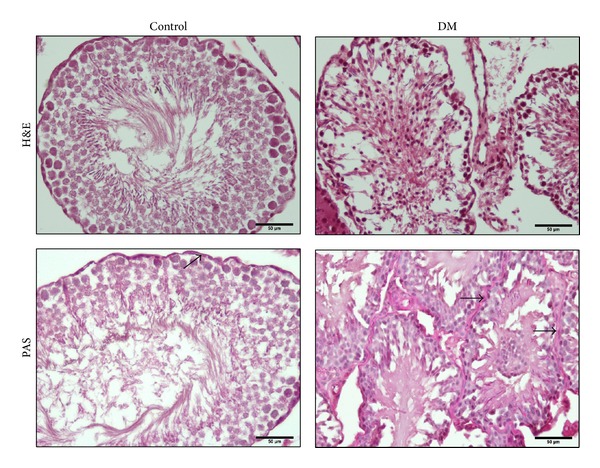
Representative photomicrographs of hematoxylin-eosin-stained and periodic-acid-Schiff-stained sections in the testes of rats. DM: diabetes mellitus. Seminiferous tubule basale membrane thickness increased in the diabetic group (arrows).

Figure 3 demonstrates histological findings of each group. Seminiferous tubules and interstitium in testicular tissue were found to be normal in the control animals. Germinal cells were found to be degenerated and disorganized and also reduced in number in diabetic rats.  Table 1 shows the comparison of the histologic changes between control and diabetic groups. In diabetic rats, the MSTD value and Johnsen's score were significantly low. Diabetic condition appeared to have impaired spermatogenetic process which was also reflected by a decrease in the mean testicular score. In diabetic group, STBM was found to be significantly thickened compared to the control group (*P* < 0.05).

**Figure 4:**
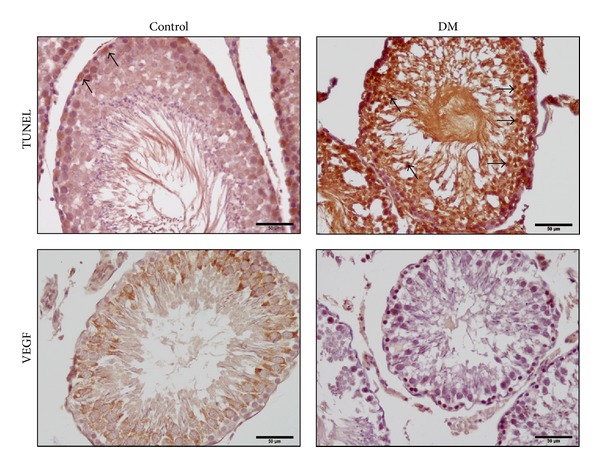
Representative photomicrographs of TUNEL+ and VEGF+ cells in the testes of rats. DM: diabetes mellitus. The number of TUNEL+ cells increased in the diabetic group (arrows).

Apoptosis and VEGF immunoreactivity are shown in Figure 4. More TUNEL-positive cells were found in diabetes group compared to control group. Quantification and statistical analysis of the TUNEL staining showed that the number of TUNEL-positive cells was significantly increased in diabetic group compared to control group (Table 1). As evidenced in the representative photographs which show VEGF expression, VEGF reactivity appears to be more prominent in control group compared to diabetic group. Intensity of VEGF immunoreactivity was scored higher in control group compared to diabetic group (Table 1).

In correlation analysis, a very strong positive correlation was found between VEGF and NGF-*β* levels (*r* = 0.925, *P* = 0.0001) (Figure 2(c)); very strong negative correlation was found between TUNEL+ cells and VEGF levels (*r* = −0.871, *P* = 0.0001) and also TUNEL+ cells and NGF-*β* levels (*r* = −0.912, *P* = 0.0001).

## 4. Discussion

Findings suggest that, in diabetic rats, the number of TUNEL-positive cells was found to be increased, seminiferous tubules were found to be impaired, spermatogenic cell series were found to be lost and associated with all these findings, and VEGF and NGF-*β* levels were found to be decreased. To our knowledge, this is the first study that investigated the correlation of diabetic process and testicular VEGF and NGF-*β* levels in rats.

It is already known that diabetes mellitus plays role in the etiology of testicular dysfunction (apoptotic cell death and atrophy of the seminiferous tubules, decreased tubule diameters, and reduction of spermatogenetic cell series) [3, 5, 6]. Major morphological indicators of spermatogenic failure are seminiferous tubules atrophy and spermatogenic cell loss [17, 18].

Johnsen's score is used to determine the presence of testicular damage [19]. In our study, tubule diameters and spermatogenetic cell series were found to be decreased in diabetic testis. Another important finding was the increase in TUNEL-positive cells in the germinal epithelium which was consistent with germinal cell apoptotic process. Apoptosis plays an important role in the pathogenesis of testicular dysfunction in diabetes [3, 17]. Several factors are known to cause apoptotic cell death such as oxidative stress and decreased neurotrophic or angiotrophic factors [3, 20, 21].

The neurotrophic factors such as NGF and VEGF are known regulators of growth, proliferation, differentiation, and survival of neurons [8, 10]. *β*-NGF is found in the nervous system and in testicular tissue [11, 12] and is known to be involved in sperm development. High levels of NGF were found in epididymal head and body (maturation of sperms occurs in epididymis). The decrease in NGF level is most likely a result of germ cell atrophy [22, 23]. In addition, Sertoli cells, spermatocytes, and early spermatids are known to synthesize NGF [22] which is involved in sperm motility and acrosome reaction [14]. In our study, NGF levels were found to be decreased in the diabetic testis.

VEGF is an angiotrophic and neurotrophic factor that mediates angiogenesis and supports endothelial cell and neuronal survival [24, 25]. VEGF is important for spermatogenesis. VEGF receptors are located on interstitial cells and seminiferous tubules [26–28]. VEGF supports proliferation and survival of germ cells [29], regulates microcirculation [27, 30], and modulates endothelial permeability in testis [31]. In addition, VEGF facilitates the transport of endocrine hormones and nutritional components [32] and oxygen [33]. Tunçkiran et al. showed that VEGF administration decreased apoptosis in testicular germ cells [34].

The expression of the VEGF is associated with the expression of NGF [35]. Topical NGF supplementation increases VEGF levels, modulates NGF receptors, and decreases apoptosis [36, 37]. NGF also is known to induce VEGF upregulation [38]. In our study, correlation between VEGF and NGF-*β* levels was found to be significant.

In conclusion, our evidence suggests that testicular VEGF and NGF-*β* levels are decreased in diabetes which is associated with increased apoptosis and testicular damage. We are suggesting that testicular VEGF and NGF-*β* levels can be helpful as biomarkers for diabetic testicular damage. Further human and animal studies are needed to determine the degree of testicular damage and its correlations with neurotrophic factors following streptozotocin injection in rats.

## Figures and Tables

**Table 1:** Seminiferous tubule diameter (STD), thickness of STBM, Johnsen's scores, TUNEL-positive cells, and VEGF-positive cells in the testes of rats.

	Micrometer	Micrometer	Johnsen's score	TUNEL+ cell	VEGF+ cell
Control	377.3 ± 12.4	4.6 ± 0.2	9.2 ± 0.4	6.5 ± 0.9	2.7 ± 0.2
DM	221.4 ± 14.8*	6.4 ± 0.2*	4.8 ± 0.4*	68.2 ± 1.3*	1.2 ± 0.2*

*P < 0.004 compared with control group. DM: diabetes mellitus.

## References

[1] Shaw JE, Sicree RA, Zimmet PZ (2010). Global estimates of the prevalence of diabetes for 2010 and 2030. *Diabetes Research and Clinical Practice*.

[2] Aksu I, Baykara B, Kiray M (2013). Serum IGF-1 levels correlate negatively to liver damage in diabetic rats. *Biotechnic & Histochemistry*.

[3] Guneli E, Tugyan K, Ozturk H, Gumustekin M, Cilaker S, Uysal N (2008). Effect of melatonin on testicular damage in streptozotocin-induced diabetes rats. *European Surgical Research*.

[4] Uysal N, Yalaz G, Acikgoz O, Gonenc S, Kayatekin BM (2005). Effect of L-carnitine on diabetogenic action of streptozotocin in rats. *Neuroendocrinology Letters*.

[5] Oksanen A (1975). Testicular lesions of streptozotocin diabetic rats. *Hormone Research*.

[6] Sexton WJ, Jarow JP (1997). Effect of diabetes mellitus upon male reproductive function. *Urology*.

[7] Ebisch IMW, Thomas CMG, Wetzels AMM, Willemsen WNP, Sweep FCGJ, Steegers-Theunissen RPM (2008). Review of the role of the plasminogen activator system and vascular endothelial growth factor in subfertility. *Fertility and Sterility*.

[8] Leung DW, Cachianes G, Kuang W-J, Goeddel DV, Ferrara N (1989). Vascular endothelial growth factor is a secreted angiogenic mitogen. *Science*.

[9] Reddy N, Kasukurthi KB, Mahla RS, Pawar RM, Goel S (2012). Expression of vascular endothelial growth factor (VEGF) transcript and protein in the testis of several vertebrates, including endangered species. *Theriogenology*.

[10] Kaplan DR, Miller FD (2000). Neurotrophin signal transduction in the nervous system. *Current Opinion in Neurobiology*.

[11] Djakiew D, Pflug B, Dionne C, Onoda M (1994). Postnatal expression of nerve growth factor receptors in the rat testis. *Biology of Reproduction*.

[12] Jin W, Arai KY, Shimizu K (2006). Cellular localization of NGF and its receptors trkA and p75LNGFR in male reproductive organs of the Japanese monkey, Macaca fuscata fuscata. *Endocrine*.

[13] Schteingart HF, Meroni SB, Cánepa DF, Pellizzari EH, Cigorraga SB (1999). Effects of basic fibroblast growth factor and nerve growth factor on lactate production, *γ*-glutamyl transpeptidase and aromatase activities in cultured sertoli cells. *European Journal of Endocrinology*.

[14] Jin W, Tanaka A, Watanabe G, Matsuda H, Taya K (2010). Effect of NGF on the motility and acrosome reaction of golden hamster spermatozoa in vitro. *Journal of Reproduction and Development*.

[15] Zhang L, Wang H, Yang Y (2013). NGF induces adult stem Leydig cells to proliferate and differentiate during Leydig cell regeneration. *Biochemical and Biophysical Research Communications*.

[16] Aksu I, Ates M, Baykara B (2012). Anxiety correlates to decreased blood and prefrontal cortex IGF-1 levels in streptozotocin induced diabetes. *Neuroscience Letters*.

[17] Cai L, Chen S, Evans T, Deng DX, Mukherjee K, Chakrabarti S (2000). Apoptotic germ-cell death and testicular damage in experimental diabetes: prevention by endothelin antagonism. *Urological Research*.

[18] Cameron DF, Murray FT, Drylie DD (1985). Interstitial compartment pathology and spermatogenic disruption in testes from impotent diabetic men. *Anatomical Record*.

[19] Kehinde EO, Anim JT, Mojiminiyi OA (2005). Allopurinol provides long-term protection for experimentally induced testicular torsion in a rabbit model. *British Journal Urology International*.

[20] Amaral S, Oliveira PJ, Ramalho-Santos J (2008). Diabetes and the impairment of reproductive function: possible role of mitochondria and reactive oxygen species. *Current Diabetes Reviews*.

[21] Uysal N, Sisman AR, Dayi A (2011). Maternal exercise decreases maternal deprivation induced anxiety of pups and correlates to increased prefrontal cortex BDNF and VEGF. *Neuroscience Letters*.

[22] Ayer-LeLievre C, Olson L, Ebendal T, Hallbook F, Persson H (1988). Nerve growth factor mRNA and protein in the testis and epididymis of mouse and rat. *Proceedings of the National Academy of Sciences of the United States of America*.

[23] Nylen P, Ebendal T, Eriksdotter-Nilsson M (1989). Testicular atrophy and loss of nerve growth factor-immunoreactive germ cell line in rats exposed to n-hexane and a protective effect of simultaneous exposure to toluene or xylene. *Archives of Toxicology*.

[24] Ferrara N (2000). VEGF: an update on biological and therapeutic aspects. *Current Opinion in Biotechnology*.

[25] Nico B, Mangieri D, Benagiano V, Crivellato E, Ribatti D (2008). Nerve growth factor as an angiogenic factor. *Microvascular Research*.

[26] Bott RC, McFee RM, Clopton DT, Toombs C, Cupp AS (2006). Vascular endothelial growth factor and kinase domain region receptor are involved in both seminiferous cord formation and vascular development during testis morphogenesis in the rat. *Biology of Reproduction*.

[27] Ergün S, Kiliç N, Fiedler W, Mukhopadhyay AK (1997). Vascular endothelial growth factor and its receptors in normal human testicular tissue. *Molecular and Cellular Endocrinology*.

[28] Korpelainen EI, Karkkainen MJ, Tenhunen A (1998). Overexpression of VEGF in testis and epididymis causes infertility in transgenic mice: evidence for nonendothelial targets for VEGF. *Journal of Cell Biology*.

[29] Caires KC, De Avila J, McLean DJ (2009). Vascular endothelial growth factor regulates germ cell survival during establishment of spermatogenesis in the bovine testis. *Reproduction*.

[30] Kaur Anand RJ, Paust H-J, Altenpohl K, Mukhopadhyay AK (2003). Regulation of vascular endothelial growth factor production by Leydig cells in vitro: the role of protein kinase A and mitogen-activated protein kinase cascade. *Biology of Reproduction*.

[31] Connolly DT, Heuvelman DM, Nelson R (1989). Tumor vascular permeability factor stimulates endothelial cell growth and angiogenesis. *Journal of Clinical Investigation*.

[32] Sone H, Deo BK, Kumagai AK (2000). Enhancement of glucose transport by vascular endothelial growth factor in retinal endothelial cells. *Investigative Ophthalmology and Visual Science*.

[33] Kilic S, Lortlar N, Bardakci Y (2009). Caspase-3 and VEGF immunopositivity in seminiferous tubule germ cells in cases of obstructive and non-obstructive azoospermia in smokers versus non-smokers. *Journal of Assisted Reproduction and Genetics*.

[34] Tunçkiran A, Çayan S, Bozlu M, Yilmaz N, Acar D, Akbay E (2005). Protective effect of vascular endothelial growth factor on histologic changes in testicular ischemia-reperfusion injury. *Fertility and Sterility*.

[35] Campos X, Muñoz Y, Selman A (2007). Nerve growth factor and its high-affinity receptor trkA participate in the control of vascular endothelial growth factor expression in epithelial ovarian cancer. *Gynecologic Oncology*.

[36] Graiani G, Emanueli C, Desortes E (2004). Nerve growth factor promotes reparative angiogenesis and inhibits endothelial apoptosis in cutaneous wounds of Type 1 diabetic mice. *Diabetologia*.

[37] Manni L, Antonelli A, Costa N, Aloe L (2005). Stress alters vascular-endothelial growth factor expression in rat arteries: role of nerve growth factor. *Basic Research in Cardiology*.

[38] Nakamura K, Tan F, Li Z, Thiele CJ (2011). NGF activation of TrkA induces vascular endothelial growth factor expression via induction of hypoxia-inducible factor-1*α*. *Molecular and Cellular Neuroscience*.

